# Risk factors associated with severe progression of Parkinson’s disease: random forest and logistic regression models

**DOI:** 10.3389/fneur.2025.1550789

**Published:** 2025-04-07

**Authors:** Jie Tan, E. Huang, Yang Hao, Hongping Wan, Qian Zhang

**Affiliations:** ^1^Department of Neurology, Hubei No. 3 People’s Hospital of Jianghan University, Wuhan, China; ^2^Support Centre, Hubei No. 3 People’s Hospital of Jianghan University, Wuhan, China

**Keywords:** Parkinson’s disease, random forest, logistic regression, risk factors, disease progression

## Abstract

**Background and aim:**

Parkinson’s disease (PD) is a neurodegenerative disorder with significant variability in disease progression. Identifying clinical and environmental risk factors associated with severe progression is essential for early diagnosis and personalized treatment. This study evaluates the performance of Random Forest (RF) and Logistic Regression (LR) models in forecasting the major risk factors associated with severe PD progression.

**Methods:**

We performed a retrospective analysis of 378 PD patients (aged 40–75 years) with at 2 years of follow-up. The dataset included patient demographics, clinical features, medication history, comorbidities, and environmental exposures. The data were randomly split into a training group (70%) and a validation group (30%). Both the RF and LR models were trained on the training set, and performance was assessed through accuracy, sensitivity, specificity, and the Area Under the Curve (AUC) derived from ROC analysis.

**Results:**

Both models identified similar risk factors for severe PD progression, including older age, tremor-dominant motor subtype, long-term levodopa use, comorbid depression, and occupational pesticide exposure. The RF model outperformed the LR model, achieving an AUC of 0.85, accuracy of 82%, sensitivity of 79%, and specificity of 85%. In comparison, the LR model had an AUC of 0.78, accuracy of 76%, sensitivity of 74%, and specificity of 79%. ROC analysis showed that while both models could distinguish between slow and rapid disease progression, the RF model had stronger discriminatory power, particularly for identifying high-risk patients.

**Conclusion:**

The RF model provides better predictive accuracy and discriminatory power compared to Logistic Regression in identifying risk factors for severe PD progression. This study highlights the potential of machine learning techniques like Random Forest for early risk stratification and personalized management of PD.

## Introduction

Parkinson’s Disease (PD) is a degenerative neurological condition that progressively impairs motor functions, with common symptoms such as tremors, rigidity, bradykinesia, and postural instability. Over time, the severity and progression of these symptoms can vary widely between patients, making it challenging to predict the course of the disease. While some patients experience relatively slow progression, others undergo rapid motor and cognitive decline, significantly affecting their quality of life. Identifying the factors associated with severe PD progression is crucial for early diagnosis, targeted treatment, and improved patient management (1–3).

The pathophysiology of PD is complex, with various genetic, clinical, and environmental factors potentially influencing disease progression. However, the precise role of these factors remains unclear. Clinical assessments, such as the Unified Parkinson’s Disease Rating Scale (UPDRS), are commonly used to track the progression of symptoms, but they fail to capture the full complexity of disease trajectory. Additionally, environmental exposures (such as pesticide use) and comorbid conditions (like depression or sleep disturbances) have been suggested as potential contributors to more severe progression, though their predictive value remains uncertain (4–7). Traditional statistical methods, such as logistic regression, have been widely used to investigate the factors influencing PD progression, but they often fall short when it comes to capturing complex, nonlinear relationships between variables. These methods, while useful for establishing baseline associations, lack the flexibility to model intricate interactions in large datasets. For example, a study by Dadu et al. (8). found that the presence of cognitive impairment and motor subtype were predictive of disease severity, but the accuracy of their logistic regression model in distinguishing between slow and fast progressors was limited. This limitation emphasizes the need for more advanced predictive models that can handle complex data and improve predictive accuracy.

In recent years, machine learning techniques such as Random Forest (RF) have gained prominence in medical research due to their ability to process large datasets and uncover hidden patterns. Random Forest, a robust ensemble learning method, has been shown to outperform traditional statistical approaches in several domains, including predicting disease outcomes and patient risk stratification (9–11). In the context of PD, RF models have demonstrated strong predictive capabilities, especially when dealing with heterogeneous data from clinical, environmental, and demographic sources. For instance, Byeon (12). employed classifier ensembles to predict rapid eye movement sleep behavior disorder in PD patients and discovered that the model could help identify individuals who would benefit from video polysomnography screening.

This study seeks to assess and compare the effectiveness of Random Forest and Logistic Regression models in predicting the risk of severe PD progression. By harnessing the advantages of these advanced methods, the goal is to develop a more precise and dependable predictive tool that could aid clinicians in identifying high-risk patients early in the disease, ultimately facilitating more personalized management approaches and enhancing patient outcomes.

## Materials and methods

### Study design and data collection

A retrospective analysis was conducted on 378 patients diagnosed with PD at Hubei No. 3 People’s Hospital of Jianghan University between January 2018 and December 2022. The dataset included clinical data collected during routine follow-ups, including patient demographics, clinical features, medical history, comorbidities, medication usage, and environmental factors (e.g., pesticide exposure). Patient data were collected at two time points: baseline (at the time of diagnosis/registration) and a two-year follow-up assessment. Of these 268 patients, 112 experienced severe disease progression as defined by rapid deterioration in motor and cognitive functions. Inclusion criteria comprised (1) diagnosed PD, (2) Age between 40 and 75 years, (3) At least 2 years of follow-up data, and (4) No comorbidities that could significantly alter PD progression. Exclusion criteria included (1) Other neurodegenerative diseases, (2) Insufficient follow-up data, (3) Missing key clinical or demographic information, and (4) Incomplete medical history. This study was carried out in compliance with the Declaration of Helsinki and received approval from the Institutional Review Board (IRB) at Hubei No. 3 People’s Hospital of Jianghan University. Patient confidentiality and data privacy were rigorously upheld throughout the study.

### Definition of PD and disease progression

PD was diagnosed according to the 2015 Movement Disorder Society Clinical Diagnostic Criteria (MDS-PD) (1). The diagnosis required the presence of bradykinesia (slowness of movement) in combination with at least one of the following features: resting tremor or rigidity. Additional supportive criteria included responsiveness to dopaminergic therapy and the absence of atypical parkinsonian syndromes. All diagnoses were confirmed by neurologists specializing in movement disorders, and patients with secondary or atypical parkinsonism (such as multiple system atrophy or progressive supranuclear palsy) were excluded from the study.

Disease Progression in PD refers to the gradual worsening of symptoms over time. The progression of PD varies widely among individuals, with some patients experiencing a slow decline in motor and cognitive functions, while others experience rapid deterioration. Severe progression of PD was defined based on motor and cognitive deterioration over a two-year follow-up period. The following clinical criteria were used:

Motor Function Deterioration: A ≥ 10-point increase in the Movement Disorder Society-Unified PD Rating Scale (MDS-UPDRS Part III) score compared to baseline, indicating a significant worsening of motor symptoms; Cognitive Decline: A ≥ 3-point reduction in the Montreal Cognitive Assessment (MoCA) score over 2 years, suggesting a clinically relevant decline in cognitive function. Overall Classification: Patients meeting either or both of the above criteria were classified as experiencing severe disease progression. These thresholds were based on established studies demonstrating that a ≥ 10-point increase in MDS-UPDRS Part III is indicative of meaningful motor decline in PD, while a ≥ 3-point MoCA decline has been associated with early cognitive deterioration in PD cohorts. Disease progression was evaluated at baseline and at the two-year follow-up visit to ensure consistency.

### Definition

Levodopa Use: In this study, “levodopa use” was defined as the regular intake of levodopa medication for at least six consecutive months at any point during the follow-up period. Patients who had never used levodopa or had a history of discontinuation before 6 months were classified as non-users.

Motor Subtype Classification: PD motor subtypes were categorized using the Movement Disorder Society (MDS) criteria, which classify patients into three groups: Tremor-Dominant (TD): Predominance of resting tremor over rigidity and bradykinesia, with a tremor-to-non-tremor ratio ≥ 1.5; Akinetic-Rigid (AR)/Postural Instability-Gait Difficulty (PIGD): Predominance of rigidity and bradykinesia, or significant postural instability and gait disturbance.

Mixed Type: Patients exhibiting overlapping symptoms without a clear predominance of tremor or rigidity.

MRI Abnormality: Brain MRI findings were evaluated for structural changes commonly associated with neurodegeneration in PD. MRI abnormalities were defined based on the presence of: White Matter Hyperintensities (WMH): Moderate to severe WMH graded using the Fazekas scale (≥2) on T2-FLAIR images.

Cortical Atrophy: Defined by significant volume loss in the frontal or temporal lobes as per neuroradiologist assessment.

Basal Ganglia Iron Deposition: Evaluated using susceptibility-weighted imaging (SWI) and defined as abnormal hypointensities suggestive of excessive iron accumulation.

### Variable selection and multicollinearity assessment in logistic regression

Candidate predictors were first screened using univariate logistic regression, with variables meeting a *p*-value threshold of <0.1 considered for inclusion in the multivariate model. The final multivariate logistic regression model was selected using backward stepwise selection based on the Akaike Information Criterion (AIC) to optimize model fit.

To ensure that collinearity did not distort the regression estimates, Variance Inflation Factor (VIF) analysis was conducted, and all included variables had VIF < 10, indicating no significant multicollinearity. Potential interactions between predictors were examined but were not retained in the final model due to lack of statistical significance.

### Data preprocessing

Normalization: Continuous variables such as age, disease duration, and medication dosage were normalized to a common scale to ensure uniformity across features and avoid bias in model training.

Feature Selection: We performed an extensive feature selection process using both statistical tests (e.g., chi-squared tests for categorical variables, ANOVA for continuous variables) and machine learning techniques (such as Random Forest feature importance) to identify the most influential factors associated with severe PD progression.

Data Augmentation: To address potential class imbalances, techniques such as oversampling of the minority class (patients with severe progression) were used. This ensured that the model could be trained without bias toward the majority class (slow-progressing patients).

### Dataset splitting

Prior to model training, the dataset was randomly split into a training set (70%) and an independent validation set (30%). The training set was used for model development and hyperparameter tuning, while the validation set served as an independent dataset for final model evaluation. To ensure model robustness and prevent overfitting, five-fold cross-validation was applied only to the training set. The training data was divided into five equal subsets (folds), with the model trained on four folds and validated on the remaining fold in each iteration. This process was repeated five times, and the final cross-validation performance metrics, such as AUC-ROC, were obtained by averaging results across all five iterations. After selecting the best hyperparameters through cross-validation, the final model was retrained using the entire training dataset (70%) and evaluated on the independent validation set (30%). The ROC curve for the validation set was generated using the final trained model on the independent validation data.

Training Set: 70% of the data (188 patients) was used to train the predictive models; Testing Set: 30% of the data (80 patients) was used to evaluate the models’ performance.

### Model development

We developed and compared two predictive models to assess the risk factors associated with severe PD progression. Logistic Regression was chosen as a traditional statistical method to model the relationship between predictor variables and the binary outcome (severe progression vs. slow progression). It assumes a linear relationship between the independent variables and the log odds of the dependent variable, and was used as the baseline model for comparison with more advanced machine learning techniques.

The Random Forest (RF) model, an ensemble learning method, was employed to capture nonlinear relationships and provide robustness against overfitting. The model constructs multiple decision trees during training, each using a randomly sampled subset of the data and predictors, and outputs the mode of the individual trees for classification. To enhance diversity among trees, at each split, a random subset of predictors equal to the square root of the total number of features was considered. The Gini impurity index was used as the criterion for measuring the quality of splits within each tree.

To optimize model performance, grid search with five-fold cross-validation was conducted to select the best combination of hyperparameters. The following parameters were explored:

n_estimators (number of trees): 100, 200, 500, 1,000.

max_depth (maximum tree depth): None (default), 10, 20, 30.

min_samples_split (minimum samples required to split a node): 2, 5, 10.

min_samples_leaf (minimum samples required for a leaf node): 1, 2, 5.

The final model was selected based on the highest area under the receiver operating characteristic curve (AUC-ROC) and the lowest out-of-bag (OOB) error rate, ensuring optimal predictive performance while minimizing overfitting. The selected model used n_estimators = 500, max_depth = None (allowing trees to grow fully), min_samples_split = 5, and min_samples_leaf = 2, which provided the best trade-off between accuracy and generalizability.

### Model training

Each model was trained on a dataset of 264 patients. To enhance predictive performance and avoid overfitting, hyperparameter tuning was performed using grid search and cross-validation techniques. For the Random Forest model, grid search was employed to explore various parameter combinations, such as the number of trees (n_estimators), maximum tree depth (max_depth), and the minimum samples required for splits and leaf nodes (min_samples_split and min_samples_leaf). The optimal parameters were selected based on performance metrics including accuracy, sensitivity, specificity, and the area under the receiver operating characteristic curve (AUC-ROC).

Cross-validation was carried out by randomly dividing the training dataset into five folds. In each iteration, four folds were used for training and one fold for validation. This process was repeated five times to ensure that every data point contributed to both training and validation, with the model’s performance averaged over the folds. Following the hyperparameter optimization, both Logistic Regression and Random Forest models were assessed on a validation dataset comprising 114 patients. Performance was compared using key metrics such as accuracy, sensitivity, specificity, and AUC-ROC to evaluate the models’ predictive power.

By leveraging hyperparameter optimization and cross-validation, the Random Forest model was fine-tuned to identify complex relationships between the predictors and severe PD progression, while Logistic Regression provided a reliable benchmark for comparison.

### Feature importance analysis

After training, feature importance analysis was conducted using the Random Forest model, which ranks features based on their contribution to the model’s predictions. This analysis helped identify the most significant clinical and environmental factors influencing severe PD progression, providing valuable insights into the disease’s underlying risk factors and improving model interpretability.

Finally, the performance of Logistic Regression and Random Forest models were compared to identify the most effective predictive model for PD progression. By analyzing the predictive accuracy, sensitivity, specificity, and AUC of each model, this study aimed to determine the best approach for risk stratification and early intervention in PD patients.

## Results

### Baseline information for PD patients in training and validation groups

**Figure 1:**
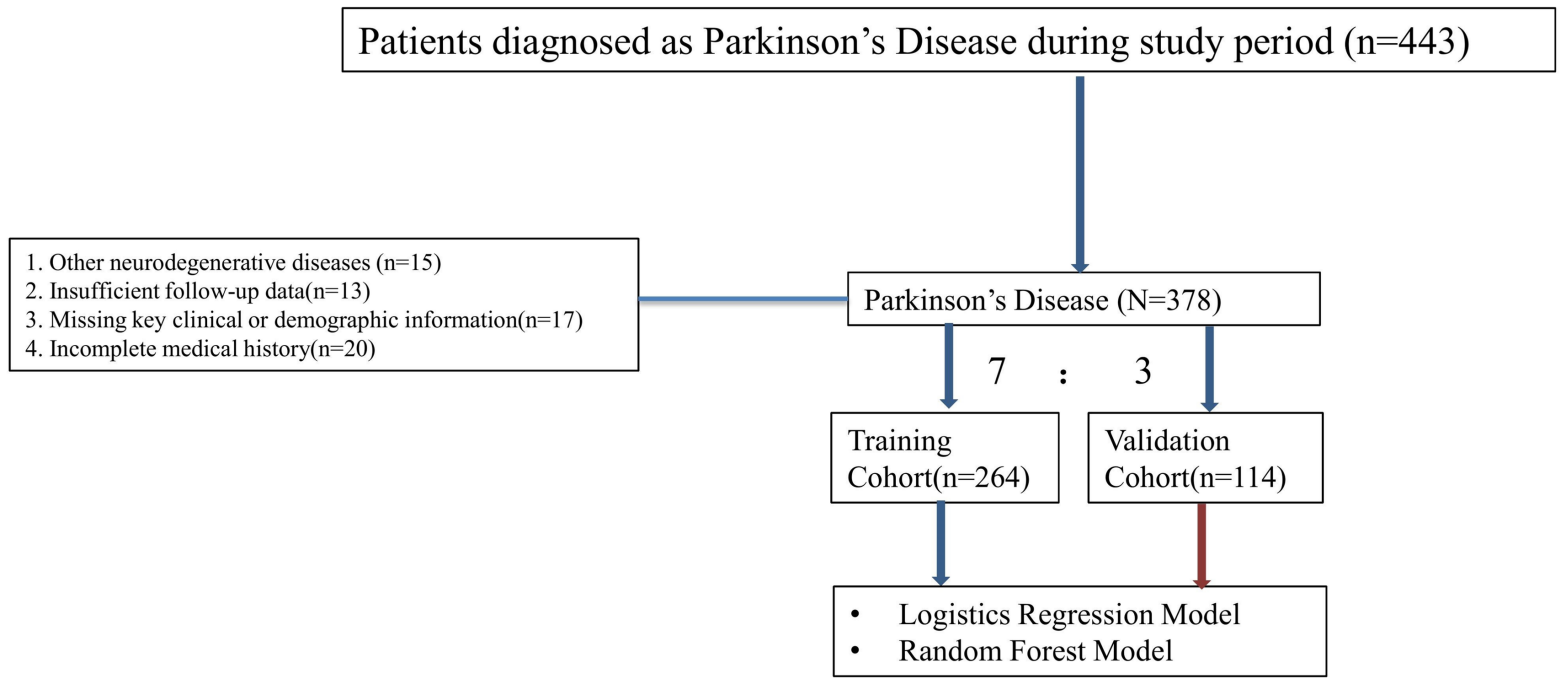
Flow chart illustrating the inclusion and exclusion criteria for patients with PD. Patients were assessed for eligibility based on specific clinical and demographic criteria.

A total of 378 patients diagnosed with PD participated in the study. The patients were randomly divided into a training cohort of 264 patients and a validation cohort of 114 patients, following a 7:3 ratio. The distribution of baseline characteristics between the two cohorts is presented in Table 1. In the training cohort, 138 patients were categorized as having severe disease progression, while 126 patients exhibited slow disease progression. In the training cohort, 191 patients (72.3%) were aged 60 years or older, with 54.9% being male and 45.1% female. In the validation cohort, 61 patients showed severe progression, and 53 patients showed slow progression. Key clinical variables such as age, BMI, disease duration, and comorbidities (e.g., hypertension, diabetes, depression) showed no significant differences between the two groups (*p* > 0.05). Additional variables, including cognitive impairment, family history, and dopamine agonist use, were also comparable across the two groups. The detailed baseline characteristics are summarized in Table 1. The comparison of baseline characteristics between included and excluded patients is shown in Supplementary Table 1, with no statistically significant differences between the two groups, indicating that the exclusion of cases had no impact on the overall study population (Figure 1).

**Table 1:** Baseline characteristics of patients with Parkinson’s disease in the training and validation cohorts (*n* = 378).

		Training cohort (*n* = 264)	Validation cohort (*n* = 114)	*P*-value
Age (%)				0.934
	<60 y	73 (27.7)	32 (28.1)	
	≥60 y	191 (72.3)	82 (71.9)	
BMI (%)				0.907
	<25	207 (78.4)	90 (78.9)	
	≥25	57 (21.6)	24 (21.1)	
Gender (%)				0.923
	Male	145 (54.9)	62 (54.4)	
	Female	119 (45.1)	52 (45.6)	
Disease progression (%)				0.825
	Severe	138 (52.3)	61 (53.5)	
	Slow	126 (47.7)	53 (46.5)	
Disease duration	Years (mean ± SD)	8.5 ± 1.6	8.6 ± 1.7	0.585
Hypertension (%)				0.609
	No	143 (54.2)	65 (57.0)	
	Yes	121 (45.8)	49 (43.0)	
Diabates (%)				0.990
	No	199 (75.4)	86 (75.4)	
	Yes	65 (24.6)	28 (24.6)	
Depression (%)				0.736
	No	183 (69.3)	81 (71.1)	
	Yes	81 (30.7)	33 (28.9)	
Cognitive impairment (%)				0.861
	No	160 (60.6)	68 (59.6)	
	Yes	104 (39.4)	46 (40.4)	
Smoking history (%)				0.908
	No	173 (65.5)	82 (66.1)	
	Yes	91 (34.5)	42 (33.9)	
Pesticide exposure (%)				0.895
	No	254 (96.2)	110 (96.5)	
	Yes	10 (3.8)	4 (3.5)	
Tremor dominant (%)				0.625
	No	109 (41.3)	44 (38.6)	
	Yes	155 (58.7)	70 (61.4)	
Rigidity dominant (%)				0.996
	No	213 (80.7)	92 (80.7)	
	Yes	51 (19.3)	22 (19.3)	
Levodopa use (%)				0.170
	No	42 (15.9)	12 (10.5)	
	Yes	222 (84.1)	102 (89.5)	
Family history (%)				0.649
	No	210 (79.5)	93 (81.6)	
	Yes	54 (20.5)	21 (18.4)	
MRI abnormalities (%)				0.532
	No	198 (75.0)	82 (71.9)	
	Yes	66 (25.0)	32 (28.1)	
Dopamine agonist use (%)				0.983
	No	134 (50.8)	58 (50.9)	
	Yes	130 (49.2)	56 (49.1)	

Definitions: Levodopa use – continuous intake for ≥ 6 months; Motor subtypes – classified according to MDS criteria (TD, AR, Mixed); MRI abnormalities – presence of WMH (Fazekas ≥ 2), cortical atrophy, or basal ganglia iron deposition. The values in parentheses are percentages unless indicated otherwise. BMI, Body Mass Index; MRI, Magnetic Resonance Imaging; SD, Standard Deviation.

### Univariate and multivariate logistic regression analysis

Univariate logistic regression analysis identified several factors significantly associated with severe PD progression. These factors included age ≥ 60 years (OR = 1.334, 95% CI: 1.125–1.649, *p* < 0.001), a history of depression (OR = 1.188, 95% CI: 1.042–1.305, *p* = 0.021), pesticide exposure (OR = 1.455, 95% CI: 1.294–1.704, *p* = 0.022), a tremor-dominant motor subtype (OR = 1.528, 95% CI: 1.254–1.905, *p* = 0.002), depression (OR = 1.188, 95% CI: 1.042–1.305, *p* = 0.021), and levodopa use (OR = 0.882, 95% CI: 0.549–0.958, *p* < 0.001). These variables were included in the multivariate logistic regression model for further analysis. The results of the multivariate analysis showed that age ≥ 60 years (OR = 1.255, 95% CI: 1.105–1.895, *p* = 0.011), pesticide exposure (OR = 1.328, 95% CI: 1.188–1.605, *p* = 0.013), depression (OR = 1.190, 95% CI: 1.084–1.304, *p* = 0.013), and a tremor-dominant motor subtype (OR = 1.755, 95% CI: 1.254–1.905, *p* = 0.006) were significant risk factors for severe PD progression. On the other hand, levodopa use (OR = 0.792, 95% CI: 0.462–0.971, *p* = 0.005) and dopamine agonist use (OR = 0.755, 95% CI: 0.519–0.855, *p* = 0.006) were associated with a reduced likelihood of severe progression. The detailed results are summarized in Table 2. In summary, age ≥ 60 years, pesticide exposure, Depression and tremor-dominant motor subtype were identified as significant risk factors for severe PD progression. Conversely, levodopa use and dopamine agonist use were found to be protective factors, associated with a reduced likelihood of severe progression (Table 2).

**Table 2:** Multivariate and univariate logistic regression analysis of patients with parkinson’s disease for identifying risk factors for disease progression.

		Univariate analysis			Multivariate analysis		
Variables		*P*	OR	95% CI	*P*	OR	95% CI
Age	≥60 y/<60 y	<0.001	1.334	1.125–1.649	0.011	1.255	1.105–1.895
BMI	≥25/<25	0.223	1.168	0.845–1.348			
Gender	Male/Female	0.119	1.281	0.799–1.826			
Hypertension	Yes/No	0.516	1.367	0.577–2.049			
Diabates	Yes/No	0.345	1.220	0.810–1.774			
Depression	Yes/No	0.021	1.188	1.042–1.305	0.013	1.190	1.084–1.304
Cognitive impairment	Yes/No	0.334	1.508	0.674–2.192			
Smoking history	Yes/No	0.220	1.340	0.658–1.995			
Pesticide exposure	Yes/No	0.022	1.455	1.294–1.704	0.013	1.328	1.188–1.605
Tremor dominant	Yes/No	0.002	1.528	1.254–1.905			
Rigidity dominant	Yes/No	0.432	1.206	0.744–1.806			
Levodopa use	Yes/No	<0.001	0.882	0.549–0.958	0.005	0.792	0.462–0.971
Family history	Yes/No	0.168	1.055	0.682–1.612			
MRI abnormalities	Yes/No	0.401	1.069	0.820–1.333			
Dopamine agonist use	Yes/No	<0.001	0.612	0.428–0.912	0.006	0.755	0.519–0.855

BMI, Body Mass Index; MRI, Magnetic Resonance Imaging.

### Model performance and ROC curve analysis

**Figure 2:**
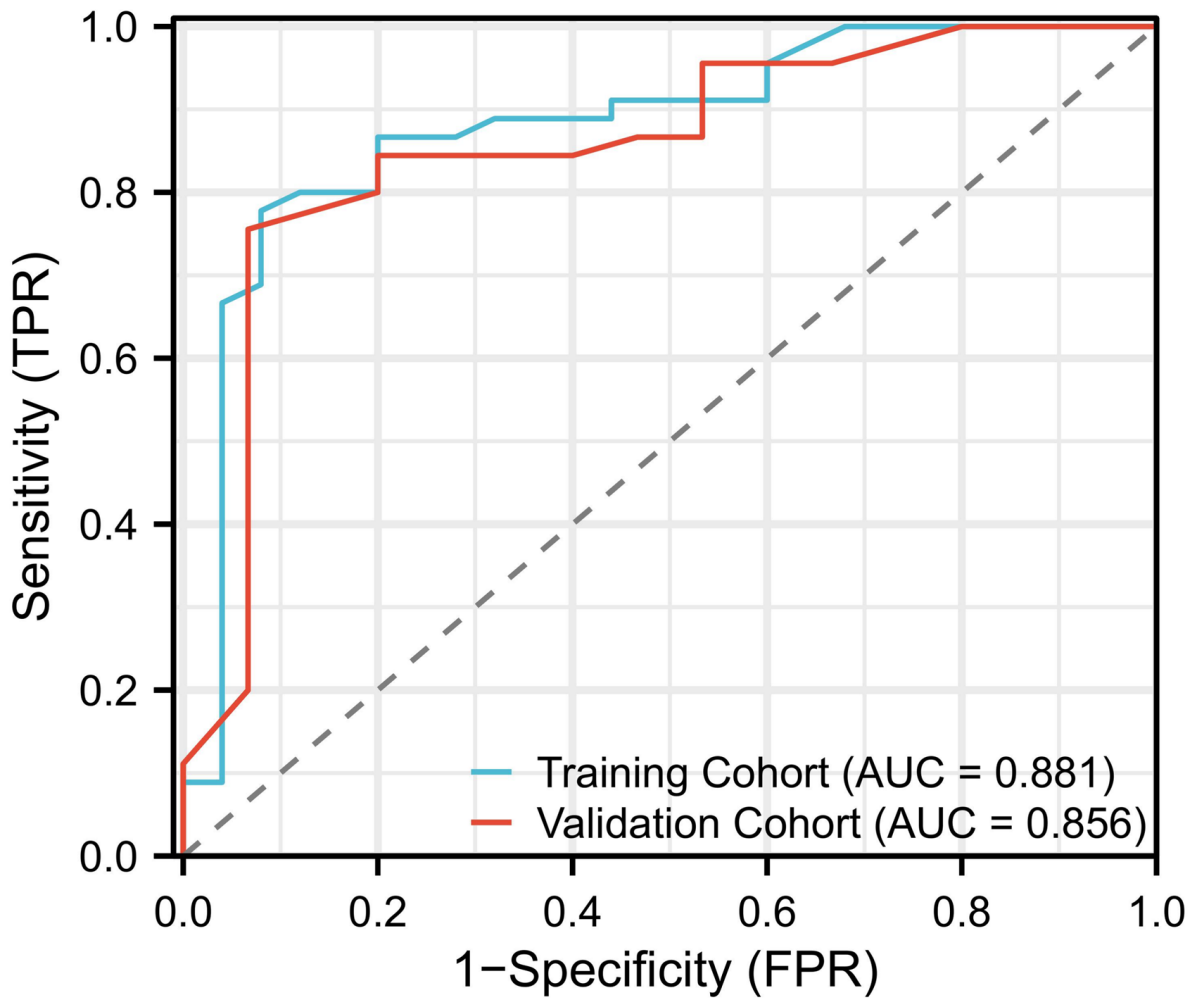
Performance of the logistic regression model, shown as the receiver operating characteristic (ROC) curve. This figure demonstrates the model’s ability to distinguish between patients with severe disease progression and those without, based on clinical features. The AUC (Area Under the Curve) of the model is shown as a measure of predictive accuracy.

**Figure 3:**
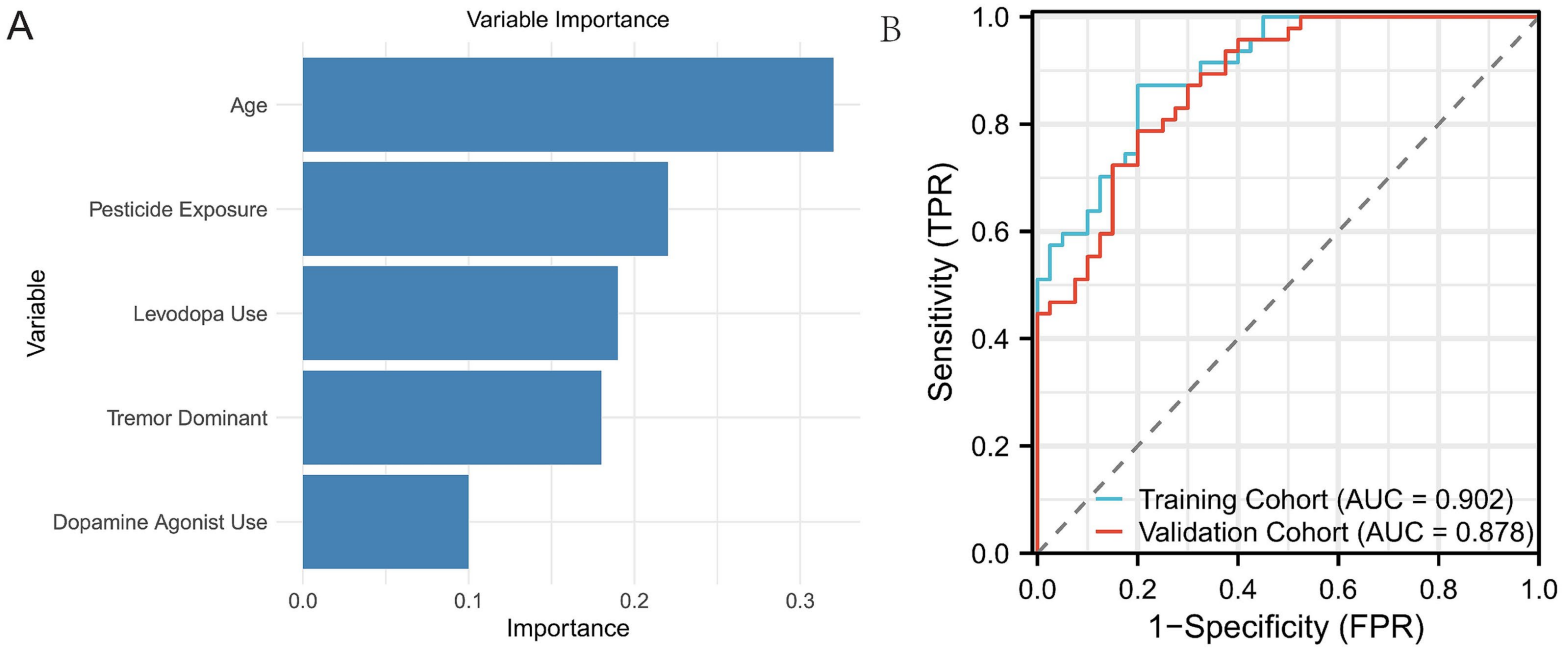
ROC curves of the Random Forest model on both the training and validation sets. **(A)** Displays the five most important variables identified by the Random Forest model based on their contribution to predicting disease progression. **(B)** Shows the ROC curve of the Random Forest model on both the training and validation sets, illustrating the model’s performance on the data.

Figure 2 displays the receiver operating characteristic (ROC) curves for the logistic regression model in both the training and validation cohorts. The blue curve represents the training cohort, achieving an area under the curve (AUC) of 0.881, indicating high predictive accuracy. The red curve represents the validation cohort, with an AUC of 0.856, demonstrating the model’s robust performance in an independent dataset. The close alignment of the curves across the cohorts suggests good generalizability of the logistic regression model. Figure 3A illustrates the variable importance for the random forest model, highlighting the most influential predictors of severe PD progression. Age was identified as the most significant variable, followed by pesticide exposure, levodopa use, tremor-dominant motor subtype, and dopamine agonist use. Figure 3B shows the ROC curves for the random forest model, with the training cohort achieving an AUC of 0.902 and the validation cohort an AUC of 0.878. These results indicate that the random forest model outperformed logistic regression in predictive accuracy, as evidenced by the higher AUC values, with feature importance scores of 0.32, 0.22, 0.19, 0.18, and 0.10, respectively (Figure 3).

### Comparison of logistic regression and random forest models

The Random Forest model outperformed the logistic regression model in both training and validation sets. The Random Forest model achieved higher AUC values (0.902 vs. 0.881 for training and 0.878 vs. 0.856 for validation) and superior predictive performance for identifying severe PD progression. Additionally, the Random Forest model demonstrated a higher F1-score in both the training set (0.902 vs. 0.840) and the validation set (0.878 vs. 0.805) compared to the logistic regression model.

In contrast, the logistic regression model showed slightly narrower confidence intervals for the AUC values in both training (0.826–0.977) and validation (0.799–0.941) sets, while the Random Forest model’s AUC confidence intervals were 0.855–0.983 for the training set and 0.824–0.961 for the validation set. Despite these differences, both models demonstrated robust performance, with the Random Forest model providing superior overall accuracy and generalizability (Table 3).

**Table 3:** Evaluation indicators for each model.

Model		F1 score	ROC-AUC	95%CI
Logistic regression				
	Training	0.840	0.881	0.826–0.977
	Validation	0.805	0.856	0.799–0.941
Random forest				
	Training	0.859	0.902	0.855–0.983
	Validation	0.843	0.878	0.824–0.961

ROC, receiver operator characteristic; AUC, area under the curve; CI, confidence interval.

## Discussion

The findings of this study indicate that machine learning models, especially the Random Forest model, surpass traditional logistic regression in predicting the severe progression of PD. Although both models demonstrated strong performance, the Random Forest model’s superior results can be attributed to its capacity to capture non-linear relationships and complex interactions between patient characteristics, which simpler models like logistic regression might overlook. Furthermore, the ensemble approach of Random Forest, which aggregates multiple decision trees, enhances its robustness and prediction accuracy, positioning it as a valuable tool for clinical decision-making (13–15).

This finding aligns with an expanding body of evidence supporting the utility of machine learning models in advancing medical predictive analytics. Machine learning techniques, such as Random Forests, have been demonstrated to outperform traditional statistical methods in identifying complex, nonlinear patterns and interactions within clinical datasets. For instance, Vaish et al. (16) explores the application of machine learning models, including Random Forest, for the early identification of prodromal PD. It highlights the strengths of ensemble methods in identifying subtle and nonlinear predictors. Similarly, Templeton et al. (17). demonstrates the effectiveness of Random Forest and other machine learning models in accurately classifying PD stages and predicting disease progression. In contrast, while logistic regression remains a valuable tool for pinpointing specific risk factors such as age, disease duration, and depression history, its linear assumptions often limit its capacity to capture the multifaceted nature of PD progression. In our study, logistic regression achieved an AUC of 0.881 in the training set, reflecting moderate predictive capability. However, it fell short in validation, where the Random Forest model excelled with an AUC of 0.902 in the training cohort and 0.878 in the validation cohort. This underscores the Random Forest model’s superior generalizability and robustness, particularly in clinical scenarios where complex interactions between predictors significantly influence outcomes. These findings support the growing consensus that machine learning models, particularly ensemble methods like Random Forests, offer transformative potential for accurately predicting the progression of diseases like Parkinson’s.

Our study identified several key risk factors associated with severe PD progression, including older age, pesticide exposure, depression, and motor subtype. Consistent with prior research, older patients showed more rapid deterioration, likely due to age-related neurodegeneration and reduced neuroplasticity (18). Pesticide exposure has been linked to increased oxidative stress and *α*-synuclein aggregation, contributing to disease progression (19). Depression, a common non-motor symptom of PD, was also associated with faster decline, potentially due to neuroinflammatory mechanisms and reduced physical activity (20). Interestingly, our study found that tremor-dominant (TD) patients progressed faster than postural instability gait difficulty (PIGD) patients, which contrasts with some previous findings suggesting that PIGD is associated with worse outcomes. This discrepancy may be due to differences in follow-up duration, cohort composition, or treatment effects, as TD patients often receive earlier dopaminergic therapy, which might temporarily alleviate symptoms but accelerate long-term decline (21, 22). Future studies with longer follow-ups and multi-center validation are needed to further explore these findings and confirm their generalizability.

PD progression is notoriously difficult to predict due to the complex, multi-faceted nature of the disease, which is influenced by a range of clinical, genetic, and environmental factors. The primary challenge in predicting the progression of PD is its highly individualized course, where different patients exhibit varying rates of motor and non-motor symptom progression. As a result, effective prediction models must account for diverse factors that may not be immediately apparent in clinical observation.

In this study, we focused on identifying and predicting the severe progression of PD, with a particular emphasis on the role of key factors such as disease duration, age, and the presence of depression. These variables have long been considered important in clinical studies on PD progression. Age at diagnosis is known to influence the progression rate, with older patients often experiencing a more rapid decline (2, 23, 24). In our Random Forest model, Age ranked as the most important variable among all predictors. Disease duration, as expected, showed a strong correlation with severity in this cohort, with longer disease duration linked to more severe symptoms. Depression history also emerged as a significant predictor. This aligns with existing research that has found depression to be not only a common non-motor symptom of PD but also a factor that may accelerate motor symptoms and cognitive decline, further complicating the disease’s trajectory. Recent research has highlighted the importance of incorporating non-motor symptoms, including cognitive dysfunction, depression, and autonomic dysfunction, into predictive models of PD progression (25–27). These non-motor symptoms are increasingly being recognized as early indicators of disease progression and may offer critical information for predicting the trajectory of the disease. This is particularly important for clinicians to manage and monitor patients effectively, as non-motor symptoms can significantly impact quality of life and may precede the worsening of motor symptoms. Moreover, the application of machine learning in PD has gained considerable attention. Random Forests and other machine learning methods can capture intricate, non-linear relationships between clinical factors that traditional models, like logistic regression, may fail to account for. The ability of Random Forest models to use various clinical, demographic, and treatment-related features to improve prediction accuracy is especially valuable in managing a heterogeneous disease like PD.

While the Random Forest model demonstrated superior predictive power compared to logistic regression, there are certain limitations to this study. The relatively small sample size of 378 patients, although statistically valid, may limit the generalizability of the results. A larger, multicenter cohort would provide a more diverse patient population, offering a clearer picture of the predictive models’ efficacy across different clinical settings.

While factors such as genetic profile, physical activity, and caffeine/nicotine intake have been discussed in the literature as potentially influencing PD progression, our study was limited by the availability of data. Genetic information was not available for the cohort, and physical activity and lifestyle factors were not consistently recorded. Despite these limitations, the findings of this study remain valuable because they highlight the predictive potential of clinical and environmental factors that are routinely collected in clinical practice, such as age, motor subtype, depression, and pesticide exposure. These factors alone were sufficient to build a model with strong predictive power for identifying patients at high risk of severe disease progression. Future studies could expand on our findings by incorporating genetic data and lifestyle factors to provide a more comprehensive understanding of disease progression. However, even without these additional factors, our study underscores the clinical applicability of using existing data for early identification and intervention in PD.

Although our study provides valuable insights into Parkinson’s disease progression using machine learning models, it is subject to certain limitations. The dataset was derived from a single-center cohort, which may limit the generalizability of our findings. While internal validation using cross-validation was performed, the lack of external validation on an independent dataset remains a limitation. While five-fold cross-validation was applied to optimize hyperparameters and assess model stability, the lack of external validation remains a limitation. The validation set was derived from the same data source, which may not fully reflect real-world variability. Future studies should validate our model using multi-center or external datasets to assess generalizability and clinical utility. Additionally, the retrospective design introduces potential selection biases, which could impact the applicability of our findings. A prospective, longitudinal study would help evaluate model performance over time in diverse populations. While our study included key clinical factors, such as disease duration and depression history, further research should explore the integration of genetic, imaging, and wearable device data. These additional features could provide real-time insights into disease progression, enhancing the predictive power of machine learning models and enabling earlier intervention in high-risk patients.

## Conclusion

This study demonstrates that machine learning models, particularly the Random Forest model, outperform traditional logistic regression in predicting the severe progression of PD. The improved predictive accuracy and robustness of Random Forest models underline their potential to aid clinicians in identifying high-risk patients early, ultimately improving patient management and treatment outcomes. Further research, especially with larger datasets, including genetic and imaging data, and the integration of continuous monitoring, could offer even more precise tools for managing PD and other complex medical conditions. Addressing the challenges related to model interpretability will be crucial to ensuring these advanced tools are adopted in clinical practice.

## Data Availability

The data analyzed in this study is subject to the following licenses/restrictions: The dataset used in this study contains sensitive patient information and is subject to the following restrictions: Confidentiality and Privacy: The dataset includes personal health information, and access is restricted to protect patient confidentiality. All data are anonymized, but direct identifiers may be present in the original dataset. Institutional Approval: Access to the dataset is only granted to researchers who have received appropriate ethical and institutional review board (IRB) approval. Requests for access will be reviewed on a case-by-case basis. Data Sharing Policies: The dataset is governed by institutional data-sharing policies, which ensure compliance with local and international data protection laws (such as GDPR or HIPAA). Any data access or sharing will be subject to the terms of these policies. Usage Limitation: The dataset can only be used for academic and research purposes. Commercial use of the data is prohibited without explicit permission from the institution. Anonymization Requirement: All data must be anonymized before sharing to prevent the identification of individual patients. The use of identifiable data is strictly prohibited outside the scope of the approved research protocol. Access to the dataset may be requested through the principal investigator or corresponding author, subject to the above restrictions and approval from the relevant ethics committee. Requests to access these datasets should be directed to zhangqian_232@163.com.
