# Supplementary material for: Risk factors associated with severe progression of Parkinson’s disease: random forest and logistic regression models
Source: Front Neurol. 2025 Apr 7;16:1550789. doi: 10.3389/fneur.2025.1550789 (PMC12009945; doi:10.3389/fneur.2025.1550789)
Supplement: Supplementary file 1 [file Table_1.DOCX]

Supplementary Table 1. Comparison of Baseline Characteristics Between Included and Excluded Patients.

|  |  | Training cohort (n=378) | Validation cohort (n=65) | P-Value |
| --- | --- | --- | --- | --- |
| Age (%) |  |  |  | 0.810 |
|  | <60 y | 105 (27.8) | 19 (29.2) |  |
|  | ≥60 y | 273 (72.2) | 46 (70.8) |  |
| BMI (%) |  |  |  | 0.766 |
|  | <25 | 297 (78.6) | 50 (76.9) |  |
|  | ≥25 | 81 (21.4) | 15 (23.1) |  |
| Gender (%) |  |  |  | 0.926 |
|  | Male | 207 (54.8) | 36 (55.4) |  |
|  | Female | 171 (45.2) | 29 (44.6) |  |
| Disease Progression (%) |  |  |  | 0.858 |
|  | Severe | 199 (52.6) | 35 (53.8) |  |
|  | Slow | 179 (47.4) | 30 (46.2) |  |
| Disease Duration | Years(mean± SD) | 8.5±1.7 | 8.5±1.7 | 1.000 |
| Hypertension (%) |  |  |  | 0.776 |
|  | No | 208 (55.0) | 37 (56.9) |  |
|  | Yes | 170 (45.0) | 28 (43.1) |  |
| Diabates (%) |  |  |  | 0.070 |
|  | No | 285 (75.4) | 49 (65.3) |  |
|  | Yes | 93 (24.6) | 26 (34.7) |  |
| Depression (%) |  |  |  | 0.880 |
|  | No | 264 (69.8) | 46 (70.8) |  |
|  | Yes | 114 (30.2) | 19 (29.2) |  |
| Cognitive Impairment (%) |  |  |  | 0.778 |
|  | No | 228 (60.3) | 38 (58.5) |  |
|  | Yes | 150 (39.7) | 27 (41.5) |  |
| Smoking History (%) |  |  |  | 0.946 |
|  | No | 255 (65.7) | 43 (66.2) |  |
|  | Yes | 133 (34.3) | 22 (33.8) |  |
| Pesticide Exposure (%) |  |  |  | 0.724 |
|  | No | 364 (96.3) | 62 (95.4) |  |
|  | Yes | 14 (3.7) | 3 (4.6) |  |
| Tremor Dominant (%) |  |  |  | 0.760 |
|  | No | 153 (40.5) | 25 (38.5) |  |
|  | Yes | 225 (59.5) | 40 (61.5) |  |
| Rigidity Dominant (%) |  |  |  | 0.897 |
|  | No | 305 (80.7) | 52 (80.0) |  |
|  | Yes | 73 (19.3) | 13 (20.0) |  |
| Levodopa Use (%) |  |  |  | 0.271 |
|  | No | 54 (14.3) | 6 (9.2) |  |
|  | Yes | 324 (85.7) | 59 (90.8) |  |
| Family History (%) |  |  |  | 0.796 |
|  | No | 303 (80.2) | 53 (81.5) |  |
|  | Yes | 75 (19.8) | 12 (18.5) |  |
| MRI Abnormalities (%) |  |  |  | 0.577 |
|  | No | 280 (74.1) | 46 (70.8) |  |
|  | Yes | 98 (25.9) | 19 (29.2) |  |
| Dopamine Agonist Use (%) |  |  |  | 0.997 |
|  | No | 192 (50.8) | 33 (50.8) |  |
|  | Yes | 186 (49.2) | 32 (49.2) |  |

The values in parentheses are percentages unless indicated otherwise.

Abbreviation:BMI: Body Mass Index; MRI: Magnetic Resonance Imaging; SD: Standard Deviation
